# QTc interval prolongation impact on in-hospital mortality in acute coronary syndromes patients using artificial intelligence and machine learning

**DOI:** 10.1186/s43044-024-00581-4

**Published:** 2024-11-13

**Authors:** Ahmed Mahmoud El Amrawy, Samar Fakhr El Deen Abd El Salam, Sherif Wagdy Ayad, Mohamed Ahmed Sobhy, Aya Mohamed Awad

**Affiliations:** 1 Cardiology Department, Faculty of Medicine, Alexandria University, Alexandria, Egypt; 2 Business Information Systems Department, College of Management and Technology, Arab Academy for Science, Technology and Maritime Transport, Alexandria, Egypt

**Keywords:** Acute coronary syndrome, Artificial intelligence, Machine learning, Data mining, In-hospital mortality

## Abstract

**Background:**

Prediction of mortality in hospitalized patients is a crucial and important problem. Several severity scoring systems over the past few decades and machine learning models for mortality prediction have been developed to predict in-hospital mortality. Our aim in this study was to apply machine learning (ML) algorithms using QTc interval to predict in-hospital mortality in ACS patients and compare them to the validated conventional risk scores.

**Results:**

This study was retrospective, using supervised learning, and data mining. Out of a cohort of 500 patients admitted to a tertiary care hospital from September 2018 to August 2020, who presented with ACS. Prediction models for in-hospital mortality in ACS patients were developed using 3 ML algorithms. We employed the ensemble learning random forest (RF) model, the Naive Bayes (NB) model and the rule-based projective adaptive resonance theory (PART) model. These models were compared to one another and to two conventional validated risk scores; the Global Registry of Acute Coronary Events (GRACE) risk score and Thrombolysis in Myocardial Infarction (TIMI) risk score. Out of the 500 patients included in our study, 164 (32.8%) patients presented with unstable angina, 148 (29.6%) patients with non-ST-elevation myocardial infarction (NSTEMI) and 188 (37.6%) patients were having ST-elevation myocardial infarction (STEMI). 64 (12.8%) patients died in-hospital and the rest survived. Performance of prediction models was measured in an area under the receiver operating characteristic curve (AUC) ranged from 0.83 to 0.93 using all available variables compared to the GRACE score (0.9 SD 0.05) and the TIMI score (0.75 SD 0.02). Using QTc as a stand-alone variable yielded (0.67 SD 0.02) with a cutoff value 450 using Bazett’s formula, whereas using QTc in addition to other variables of personal and clinical data and other ECG variables, the result was 0.8 SD 0.04. Results of RF and NB models were almost the same, but PART model yielded the least results. There was no significant difference of AUC values after replacing the missing values and applying class balancer.

**Conclusions:**

The proposed method can effectively predict patients at high risk of in-hospital mortality early in the setting of ACS using only clinical and ECG data. Prolonged QTc interval can be used as a risk predictor of in-hospital mortality in ACS patients.

## Background

Globally, acute coronary syndromes (ACS) are the main cause of mortality [1–3]. The term “ACS” refers to a group of diseases that primarily result from inadequate blood flow to the myocardium after an acute rupture or erosion of cholesterol plaque, which causes thrombus formation. ACS includes non-ST-segment elevation acute coronary syndrome (NSTE-ACS) and ST-elevation myocardial infarction (STEMI) [3, 4]. The primary complaint of individuals with ACS is chest discomfort, which also happens to be one of the most frequent reasons for ER visits [5]. For proper management of ACS patients, accurate early risk stratification of subsequent adverse cardiovascular events has an important role [4]. Many risk scores have been developed and validated for risk stratification. Among these, the Global Registry of Acute Coronary Events (GRACE) and the Thrombolysis in Myocardial Infarction (TIMI) scores are the most commonly used and advised in clinical practice guidelines. However, the requirement of some data which may not be easily accessible during the patient’s initial presentation to the ER such as troponin, which can take hours to obtain is a common limitation to early risk prediction or stratification using these traditional beneficial clinical scores [4, 6, 7].

Clinical examination, electrocardiogram (ECG) and cardiac troponin or high sensitive troponin (cTn) are the main components for early ACS diagnosis [5, 8–10]. Hence, the 12-lead ECG is readily available during early initial patient assessment in ER, provides diagnostic prediction in seconds and can serve as a low cost, noninvasive, and quick diagnostic and screening tool [11]. Among the different parameters of ECG, QTc interval can be easily calculated using Bazett’s formula.

QTc prolongation appeared to develop even before traditional ECG indicators of ischemia such as ST-segment deviation or T-wave changes [3, 12]. There is a strong association between QT interval prolongation and elevated risk of cardiovascular morbidity and mortality, as well as sudden death in general and in ACS population particularly [13, 14].

A subset of artificial intelligence known as machine learning (ML) uses algorithms to automatically acquire knowledge by finding patterns or information in data [15, 16]. ML is an emerging field in advanced computer science that has been widely used in clinical research to enhance predictive modeling and explore novel or new predictors of a particular outcome [15, 17]. Nowadays, medical professionals and health informatics use machine learning extensively for the diagnosis and prognosis of cardiovascular diseases, particularly to discover new genotypes and phenotypes in the diseases that already exist, enhance patient care, enable cost-effectiveness and reduce readmission and mortality rates [17, 18].

We hypothesized that QTc prolongation is one of the predictors of in-hospital mortality post-ACS. Therefore, we evaluated the incremental prognostic value of QT prolongation in early mortality prediction using a data mining approach.

## Methods

### Study population

A retrospective, supervised learning data mining study was conducted on data collected from 500 patients with established diagnosis of ACS; (STEMI, NSTEMI or unstable angina) according to ESC and AHA guidelines [5, 8–10]; hospitalized in a tertiary care hospital, from September 2018 to August 2020. All included patients had ECG with sinus rhythm on admission. Patients with mechanical complications of ACS, preexcitation or high-degree atrioventricular block, preexisting left bundle branch block, known valvular heart diseases or congenital heart anomalies, atrial fibrillation, cardiomyopathies, implanted pacemakers or cardiac resynchronization therapy (CRT) devices were excluded. Hypocalcemia, hypokalemia, hypomagnesaemia or use of any drug that prolong QTc interval were also excluded, as well as missing or unreadable ECGs, unreadable QT intervals.

### Study objectives

The primary objective was development of numerous prediction models, based on different ML algorithms for in-hospital mortality post-ACS, while evaluating the effects of the algorithm type on the models’ predictive performance. Secondary objective was to compare their performance with contemporary risk score (i.e., TIMI and GRACE).

### Data collection

Data of the included patients were gathered manually from hospital records in an Excel sheet due to the absence of electronic medical records (EMRs). Patients’ records included thorough information with special focus on:

A. Admission data:
A Age and gender of included patients.
B History of diabetes or hypertension.
C Past history of acute coronary syndrome, PCI or CABG.
D Special habits: alcohol intake, tobacco use; current, former or nonsmoker.
E Clinical assessment on admission: symptoms of the patients, level of consciousness, Killip class and vital signs (pulse, blood pressure, oxygen saturation SO2 and respiratory rate) with maximum and minimum values in the first 6 h and 24 h from admission.
B. 12-lead resting electrocardiogram (ECG) was done on admission with data analysis as: ventricular rate, PR interval, QRS complex duration, QT interval, QTc interval, average RR interval, any ST-segment changes and T-wave changes.
C. Laboratory data including complete blood picture (CBC), serum creatinine, serum urea, SGOT, SGPT, serum potassium (K) and sodium (Na) levels, lipid profile, PTT, INR and two rounds cardiac markers (CK-MB and troponin).
D. Echocardiography was done within the first 24 h of admission with assessment of: left ventricular function; estimated ejection fraction (EF), end-systolic and diastolic dimensions, presence of any valvular lesion and its severity, presence of resting regional wall motion abnormalities (RWMA), dimensions of left atrium (LA), interventricular septum (IVS) and inferior vena cava (IVC), estimated pulmonary artery systolic pressure (PASP), detection of pulmonary acceleration time (PAT) and measurement of tricuspid annular plane systolic excursion (TAPSE).
E. Any intervention done, either coronary angiography with or without stenting, CABG or thrombolysis.
F. Calculation of TIMI and GRACE scores for all patients.

### Study design

Three representative supervised machine learning algorithms were selected: the rule-based projective adaptive resonance theory (PART) models, the ensemble learning random forest (RF) and the Naive Bayes (NB) models. These algorithms can predict a patient’s survival based on previously learned information. The primary outcome was in-hospital mortality. The experimenter and explorer modules of Waikato Environment for Knowledge Analysis (WEKA v. 3.9.3, New-Zealand), a data mining software, were used for model development and evaluation. Predictive ability was assessed using the area (AUC) under the receiver operating characteristic curve (ROC). The mean and mode values were utilized to impute missing values for numerical and nominal variables, respectively.

Prediction models for in-hospital mortality after ACS were developed using 3 ML algorithms on all 80 variables of the above-mentioned data. A prediction model was trained and tested through stratified tenfold cross-validation (CV) for all variables first and for QTc as a stand-alone variable. Early prediction simplified model (EPM) was developed for early mortality prediction using data readily available on admission of the patients including personal data, early clinical assessment and ECG data. The EPM was trained for only 22 variables could easily be collected once admission in ER. Predictive performance was calculated and averaged based on the validation subsamples.

ACS in-hospital mortality prediction is a typical imbalanced learning problem and therefore, directly applying the standard machine learning algorithms, which rely on the assumption that samples are balanced distributed may lead to poor performance.

Resampling strategy and class balancer were used to alleviate this and adjust the class distribution of the imbalanced training data to effectively place more importance on the minority-class then run the 3ML algorithms on the data after resampling and class balancer for the three models; the full model, the QTc only and the EPM, and the results were compared.

### Comparison to TIMI and GRACE risk scores

The TIMI and GRACE risk scores were calculated for all patients without missing values or data, and their performance (AUC) was compared to the developed ML-based models.

### Statistical analysis

For parametric variables, results were expressed as mean ± SD, while for nonparametric variables, results were expressed as frequencies/percentages. The two-sided independent student t-test and the Chi-square test were used in the univariate analysis to determine the significant variables (p < 0.05). The analysis was performed using SPSS (v.20, IBM).

## Results

### Patient characteristics

Of the 500 patients included in this study, 164 (32.8%) patients presented with unstable angina, 148 (29.6%) patients with non-ST-elevation myocardial infarction (NSTEMI) and 188 (37.6%) patients were having ST-elevation myocardial infarction (STEMI). 64 (12.8%) patients died in-hospital and the rest survived. The mean age was 58.8 SD 12 and 77% were males.

The majority (61%) were treated with PCI, 8.6% were referred for CABAG and the rest were treated medically. Survivors significantly differed from non-survivors in age, previous cardiac history, smoking status, Killip class, incidence of arrhythmia, heart rate, systolic, and diastolic blood pressure, respiratory rate, QT duration and QTc interval, ST-segment deviation (Table 1).

**Table 1:** Clinical characteristics of the study population (variables of the EPM)

	Mean ± SD or %			*P* value
	Total (*n* = 500)	Dead (*n* = 64)	Alive (*n* = 436)	
Age	58.82 ± 12.04	67.56 ± 12.11	57.53 ± 11.49	< 0.001*
Gender (male)	385 (77%)	47 (73.4%)	338 (77.5%)	0.468
Smoking	346 (69.2%)	42	304	0.013*
Diabetes	250 (50%)	38	212	0.108
Hypertension	312 (62.4%)	42	270	0.568
*Prior MI*				
No	357 (71.4%)	42 (65.6%)	315 (72.2%)	MCp = 0.004*
Medical treatment	27 (5.4%)	9 (14.1%)	18 (4.1%)	
PCI	90 (18%)	8 (12.5%)	82 (18.8%)	
CABG	12 (2.4%)	4 (6.3%)	8 (1.8%)	
PCI + CABG	14 (2.8%)	1 (1.6%)	13 (3%)	
Comorbidities				
CKD	51 (10.2%)	18 (28.1%)	33 (7.6%)	MCp = < 0.001*
COPD	6 (1.2%)	1 (1.6%)	5 (1.1%)	
CVS	8 (1.6%)	0	8 (1.6%)	
PVD	3 (0.6%)	2 (3.1%)	1 (0.2%)	
*Killip class*				
I	419 (83.8%)	22 (34.4%)	397 (91.1%)	< 0.001*
II	49 (9.8%)	16 (25%)	33 (7.6%)	
III	16 (3.2%)	11 (17.2%)	5 (1.1%)	
IV	16 (3.2%)	15 (23.4%)	1 (0.2%)	
Min HR 6h	71.71 ± 14.15	80.61 ± 22.98	70.40 ± 11.83	< 0.001*
Max HR 6h	85.22 ± 18.03	104.42 ± 26.92	82.40 ± 14.35	< 0.001*
Min SBP 6h	107.72 ± 17.86	94.84 ± 32.37	109.61 ± 13.65	< 0.001*
Max SBP 6h	125.58 ± 18.46	123.13 ± 25.13	125.94 ± 17.28	0.163
Min DBP 6h	67.14 ± 11.41	57.19 ± 19.88	68.60 ± 8.69	< 0.001*
Max DBP 6h	78.54 ± 10.82	73.59 ± 14.84	79.27 ± 9.92	< 0.001*
Min respiratory rate 6h	17.08 ± 1.92	17.66 ± 2.87	17.0 ± 1.73	0.011*
Max respiratory rate 6h	20.26 ± 2.73	23.03 ± 4.71	19.85 ± 2.01	< 0.001*
Ventricular rate	79.57 ± 17.03	94.86 ± 21.78	77.33 ± 14.99	< 0.001*
PR interval (msec)	162.81 ± 25.95	170.13 ± 33.25	161.74 ± 24.56	0.048*
Average RR (msec)	781.80 ± 164.42	669.52 ± 169.79	798.29 ± 157.19	< 0.001*
QRS duration (msec)	96.23 ± 11.74	98.84 ± 13.02	95.85 ± 11.50	0.056
QT interval (msec)	375.40 ± 40.23	363.06 ± 48.82	377.22 ± 38.55	0.006*
QTcB (msec)	430.48 ± 37.17	450.94 ± 46.30	427.47 ± 34.69	< 0.001*
ST deviation	216 (43.2%)	54	162	< 0.001*

MI, myocardial infarction; PCI, percutaneous coronary intervention; CABG, coronary artery bypass graft; CKD, chronic kidney disease; COPD, chronic obstructive pulmonary disease; CVS, cerebrovascular stroke; PVD, peripheral vascular disease; min, minimum; max, maximum; HR, heart rate; SBP, systolic blood pressure; DBP, diastolic blood pressure

*results ≤ .05 are statistically significant

The in-hospital mortality had higher prevalence of patients with STEMI, CKD, higher Killip class (III and IV), elevated CK-MB, and fivefold elevated troponin, renal functions, liver functions, hemoglobin, WBCs, platelets count, LDL, TG, Na, K, number of diseased vessels, EF, ESD, EDD, RV function, presence of mitral, tricuspid, and aortic valve regurgitation, presence of pulmonary hypertension and higher TIMI and GRACE scores.

### Primary objective prediction

**Fig. 1:**
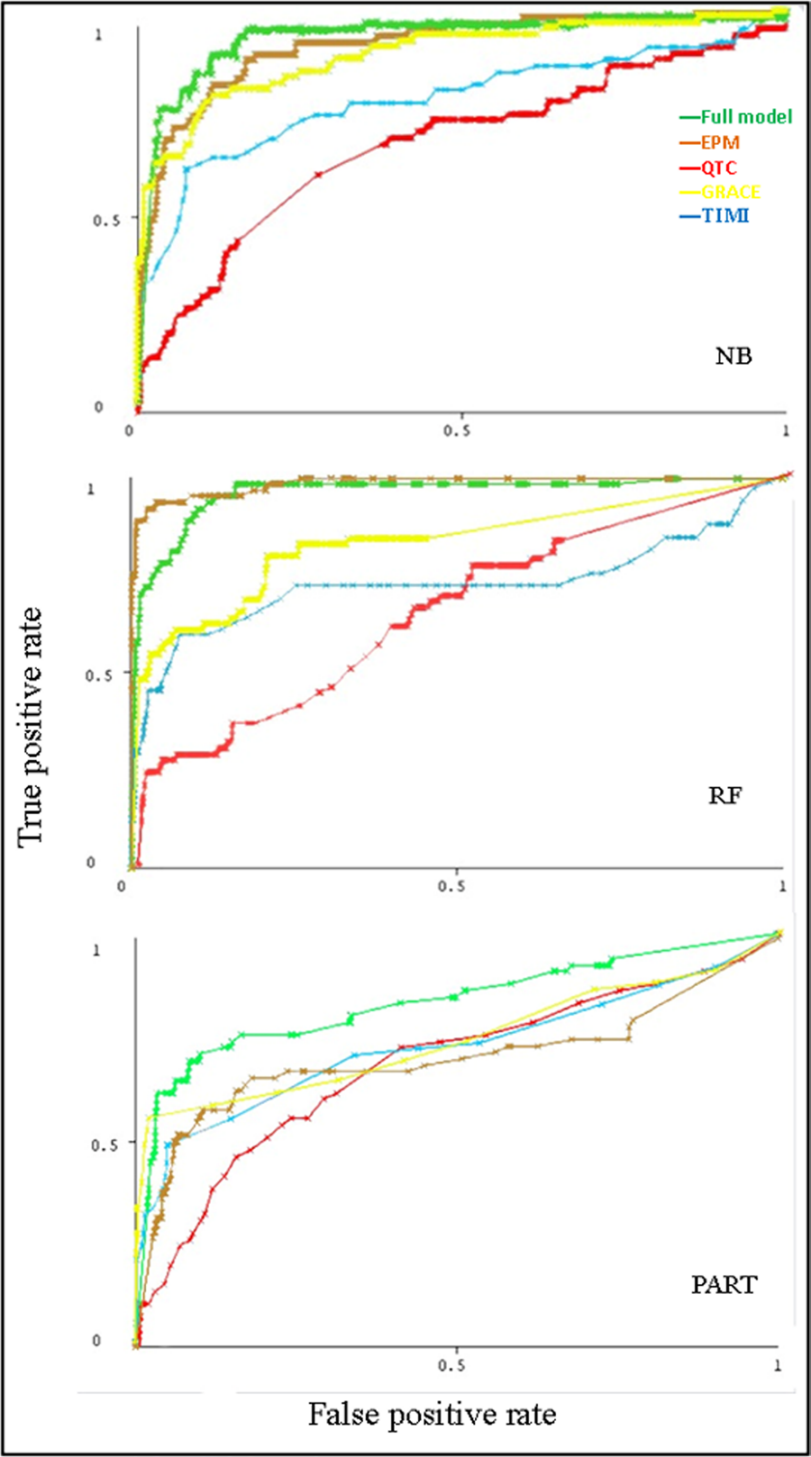
Comparison between the ROC curves performance of the 3 algorithms (NB, RF, PART) on the original dataset

Using all variables available, the prediction models for in-hospital mortality post-ACS were developed using 3 ML algorithms. The maximal predictive performance of the full model was observed with the NB algorithm (AUC = 0.93), performing similarly to RF (AUC = 0.925) and significantly better than PART algorithm (AUC = 0.82). Performance results of the EPM didn’t significantly differ from the full model for both NB algorithm (AUC = 0.913) and RF algorithm (AUC = 0.9), unlike the PART algorithm (AUC = 0.7) with lesser performance than the full model. For QTc as a stand-alone variable, the performance of the 3 ML algorithms were nearly the same; NB algorithm (AUC = 0.69), RF (AUC = 0.66) and PART algorithm (AUC = 0.67), which were significantly different from the performance of both full and EPM. The cutoff value of QTc were 450 ms, above this value the risk of in-hospital mortality was significant (Fig. 1).

**Fig. 2:**
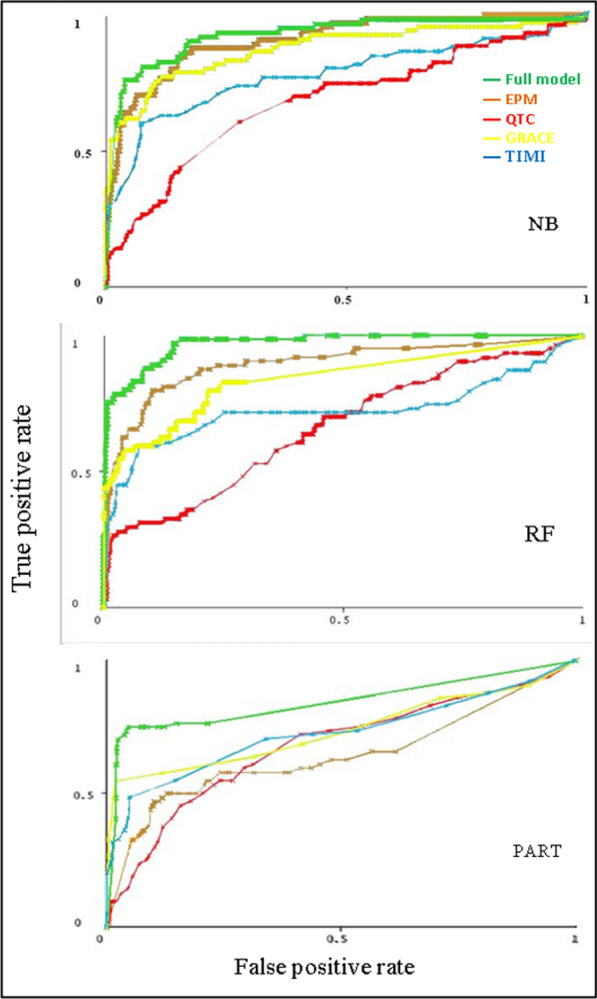
Comparison between the ROC curves of the 3 algorithms (NB, RF, PART) after replacing the missing values

After replacing the missing values for nominal and numeric attributes of the dataset with modes and means from the training data, the performance of the models was reassessed. There was no significant difference in the predictive performance of the full model after replacing the missing values from the original dataset; for NB algorithm (AUC = 0.93), RF algorithm (AUC = 0.97) and PART algorithm (AUC = 0.84). No missing values were found in the dataset of the EPM and the QTc (Fig. 2).

**Fig. 3:**
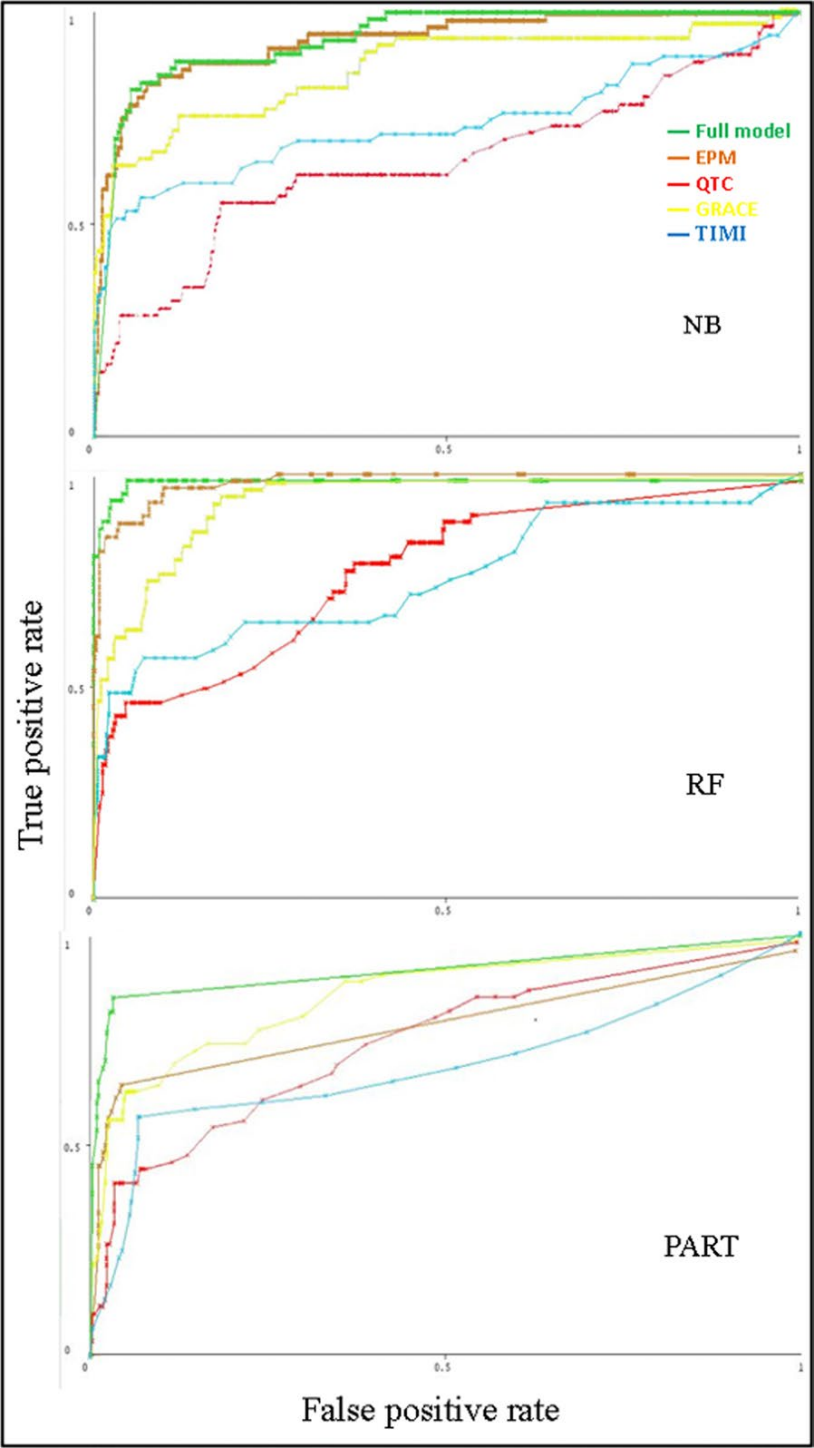
Comparison between the ROC curves performance of the 3 algorithms (NB, RF, PART) after resampling and class balancer

Resampling and class balancer were applied for each model after replacing the missing values. The observed predictive performance for the full model was nearly the same for NB (AUC = 0.94) as the original dataset, but with better performance for RF(AUC = 0.99) and PART (AUC = 0.91) algorithms. The EPM had almost the same results of the full model after resampling and class balancer for both NB (AUC = 0.93) and RF (AUC = 0.99) algorithms, but not the PART algorithm (AUC = 0.82). Performance of QTc got better than the original dataset; for NB (AUC = 0.65), RF (AUC = 0.78) and PART (AUC = 0.75) algorithms (Fig. 3).

### Comparison to TIMI and GRACE risk scores

NB and RF models had better predictive performance over the GRACE score (AUC = 0.82 SD 0.06), unlike the PART model which had similar performance compared to GRACE score. The three ML algorithms were significantly better than the TIMI risk score (AUC = 0.74 SD 0.03).

## Discussion

We have developed three ML-based prediction models for in-hospital mortality post-ACS by applying a data mining approach. Our proposed method showed superior performance to TIMI risk score, while achieving comparable performance with GRACE score. Our study’s primary goal was to create a predictive model using variables that are rapidly and readily available at the earliest possible medical contact, rather than to replace the existing risk scores.

Both NB and RF algorithms had nearly the same performance which was better than PART algorithm. Although the predictive performance of PART algorithm got better after missing value imputation and applying resampling and class balancer in contrast to the NB algorithm that had results almost similar to the original dataset.

We established risk stratification models, one for the full, one for the reduced EPM and another one for QTc variable for the purpose of predicting in-hospital mortality in patients with ACS. In the derivation cohort, the AUC of the full model was the best; this makes sense because all available variables were used in the full model. The EPM model, which only considered the demographic data, clinical assessment, vital signs and ECG, still revealed a high AUC (0.9). The application of the EPM model is practical, as only noninvasive features were needed; these data are easy to collect, do not require additional laboratory work and should therefore facilitate a simple and cost-effective workflow in a hospital setting.

ECG is the first easy, affordable and widely used bedside diagnostic modality for assessment of cardiac chest pain. The QTc interval is one of the many ECG parameters that are simple to calculate. As a result, this study assessed the QTc interval’s incremental predictive value beyond the conventional risk scores. Acute myocardial ischemia has been shown to prolong the QTc interval and increase the heterogeneity of repolarization in the myocardium [19]. Multiple possible mechanisms have been suggested, the sympathetic and neurohormonal theory is one of them. Acute myocardial ischemia changes the response of the myocardium to catecholamine or cholinergic stimulation, increased levels of catecholamines in ischemic myocardium may cause QT prolongation [20]. Another proposed mechanism is the changes associated with potassium and calcium ion channels and changes affecting the late sodium ion current occurring in the early phases of myocardial ischemia which may prolong the action potential. The other possible mechanisms are a reduction of the epicardium temperature, impedance changes and acidosis occurring early in acute myocardial infarction, so that QTc interval may be an early sign of myocardial ischemia [13, 14, 19].

Different findings have been found in epidemiological studies investigating the correlation between QTc prolongation and cardiovascular mortality [21, 22]. In a healthy population, the Framingham study did not find any correlation between baseline QTc prolongation and coronary mortality, sudden death or overall mortality. On the other hand, in a healthy population, the Cardiovascular Health Study found a correlation between a QTc > 450 ms and overall mortality. QTc > 460 ms was associated with an increased risk of twofolds for cardiac and overall mortality in a healthy population in the Strong Heart Study. In the Rotterdam Study of elderly population, there was association between QTc prolongation > 440 ms and risk of total and cardiovascular mortality [22]. A prolonged QTc interval > 458 ms in ACS patients on the ECG at admission, was an independent predictor of cardiovascular risk of mortality and recurrent ACS, according to studies by Gadelata et al. and Jiménez-Candil et al. [23, 24]. According to De Venecia et al., patients with at least one cardiac risk factor who report to the emergency room (ER) with chest pain are more likely to die within a year or have an ACS if their QTc interval is prolonged and exceeds 460 ms [22].

QTc interval prolongation has been used as a risk predictor for stratifying ACS patients in several studies, with cutoff value ranged between 440 and 450 ms [25]. Watto et al., found that STEMI patients having prolonged QTc were approximately four folds more likely to have serious adverse outcomes including ventricular arrhythmias and increased in-hospital mortality [21]. Another study reported that presence of prolonged QTc interval in admission ECG in STEMI patients was associated with an increased risk of in-hospital mortality [26]. This finding aligns with the results of another study that showed that QTc > 445 ms was an independent predictor of all-cause mortality and heart failure in patients with STEMI. A study on NSTEMI patients without early ischemic changes on admission ECG showed that QTc prolongation > 458 ms was an independent predictor of cardiovascular risk [28, 29]. In a study by Rajvanshi et al., in NSTEMI patients, prolonged QTc > 468 ms was found to have independent prognostic value superior to TIMI risk score to predict MACE (AUC = 0.684, a sensitivity of 72% and specificity of 61%) [20, 27].

A similar study to ours used ML for MACE prediction in acute MI patients using full and reduced models, with good performance of the reduced one (AUC = 0.88) which only considered age, risk factors for CAD and QTc as variables to facilitate early, simple, noninvasive risk prediction. It stated that QTc prolongation is a risk biomarker for MACE prediction [25].

Corrected QT interval is a simple, practical, affordable, independently prognostic with additive value to the existing risk predictors in ACS patients [30]. It can be routinely used in conjunction with the traditional risk scores, and newer scores may be created in the future with QTc inclusion for improved risk stratification.

### Implications for medical practice

Evaluating the importance of a treatment modality and its cost-effectiveness requires the prediction of the future event in the patient’s care plan. Moreover, identifying the risk predictors for mortality post-ACS provides clinician's and patient's crucial information, for better prognosis and improved management. Considerable progress to mortality predictive models’ specificity may reduce unnecessary admission and help in allocation of resources, which is crucial in countries with limited healthcare resources [31, 32].

There are potential applications of the EPM. First, early stratifying patients at the ER without the use of blood tests may help in quick identification of high-risk patients and, afterward, to provide them with prompt, more rapid and effective treatment, and interventions, and thus help to reduce the mortality rate. Second, some of the variables that were found in this model may offer information that may be used to update the existing ACS risk scores.

This research may result in the widespread use of machine learning–based algorithms for the creation of precise, accurate and broadly applicable risk assessment tools in cardiology clinical practice.

## Limitations of the study

The present study has some limitations. First, data were obtained from a single center, inclusion of various hospital datasets and external validation may have contributed more value and reliability. However, it identified potential variables for ACS patients and demonstrated high performance, so it may be taken into consideration in clinical practice, in conjunction with the existing risk scoring systems for improved patient safety and effectiveness. Second, it is retrospective research with possible biases in data selection and measurements. Yet, the data analysis conveys more recent practice because it is based on real world practice data. Third, we focused on in-hospital mortality which is a short-term outcome. Future models should aim to predict long-term outcomes such as recurrent MI and long-term mortality. Fourth, we used a simplified technique for missing data imputation, which is liable to bias. However, it is a general and commonly used method in practice for replacing the missing values in ML, in addition, the cohort’s missing values rate was relatively small, and the results were similar to those obtained without missing data imputation. Finally, the prediction model was built using a relatively small sample size of 500 patients with the possibility of overfitting problem. Although we applied resampling and class balancer to overcome the overfitting problem, the results showed high predictive performance almost similar to the original dataset for both RF and NB algorithms.

## Conclusion

In conclusion, we present a data mining approach for predicting post-ACS in-hospital mortality. Our results showed the feasibility and competence of ML algorithms for predictive modeling in a complex clinical scenario in the field of cardiology. So that our proposed method may be employed for risk stratification as a substitute to the traditional approach, with comparable performance to validated risk scores. In patients with ACS, QTc prolongation upon ER presentation can be used as an independent risk predictor for in-hospital mortality. This finding may offer additional value to conventional risk scores, such as TIMI and GRACE scores. Corrected QT interval may be used along with conventional risk scores and newer scores may be developed with QTc inclusion in the future for better risk stratification.

## Data Availability

The datasets used and/or analyzed during the current study are available from the corresponding author on reasonable request.

## Abbreviations

ACS: Acute coronary syndromes
CABG: Coronary artery bypass grafting
CAD: Coronary artery disease
CKD: Chronic kidney disease
CRT: Cardiac resynchronization therapy
cTn: Cardiac troponin
ECG: Electrocardiogram
EDD: End-diastolic dimension
EF: Ejection fraction
EMR: Electronic medical records
ESD: End-systolic dimension
EPM: Early prediction simplified model
ER: Emergency room
GRACE: Global Registry of Acute Coronary Events
ML: Machine learning
NB: Naive Bayes model
NSTEMI: Non-ST-elevation myocardial infarction
PART: Projective adaptive resonance theory model
PCI: Percutaneous coronary intervention
RF: Random forest model
STEMI: ST-elevation myocardial infarction
TIMI: Thrombolysis in Myocardial Infarction
